# Multi-Seasonal Nitrogen Recoveries from Crop Residue in Soil and Crop in a Temperate Agro-Ecosystem

**DOI:** 10.1371/journal.pone.0133437

**Published:** 2015-07-20

**Authors:** Guoqing Hu, Xiao Liu, Hongbo He, Wei Zhang, Hongtu Xie, Yeye Wu, Jiehua Cui, Ci Sun, Xudong Zhang

**Affiliations:** 1 State Key Laboratory of Forest and Soil Ecology, Institute of Applied Ecology, Chinese Academy of Sciences, Shenyang, 110164, China; 2 University of Chinese Academy of Sciences, Beijing, 100049, China; 3 National Field Observation and Research Station of Shenyang Agro-ecosystems, Shenyang, 110016, China; Beijing Normal University, CHINA

## Abstract

In conservation tillage systems, at least 30% of the soil surface was covered by crop residues which generally contain significant amounts of nitrogen (N). However, little is known about the multi-seasonal recoveries of the N derived from these crop residues in soil-crop systems, notably in northeastern China. In a temperate agro-ecosystem, ^15^N-labeled maize residue was applied to field surfaces in the 1^st^ year (2009). From the 2^nd^ to 4^th^ year (2010-2012), one treatment halted the application of maize residue, whereas the soil in the second treatment was re-applied with unlabeled maize residue. Crop and soil samples were collected after each harvest, and their ^15^N enrichments were determined on an isotope ratio mass spectrometer to trace the allocation of N derived from the initially applied maize residue in the soil-crop systems. On average, 8.4% of the maize residue N was recovered in the soil-crop in the 1^st^ year, and the vast majority (61.9%-91.9%) was recovered during subsequent years. Throughout the experiment, the cumulative recovery of the residue N in the crop increased gradually (18.2%-20.9%), but most of the residue N was retained in the soil, notably in the 0-10 cm soil layer. Compared to the single application, the sequential residue application significantly increased the recovery of the residue N in the soil profile (73.8% vs. 40.9%) and remarkably decreased the total and the initially applied residue derived mineral N along the soil profile. Our results suggested that the residue N was actively involved in N cycling, and its release and recovery in crop and soil profile were controlled by the decomposition process. Sequential residue application significantly enhanced the retention and stabilization of the initially applied residue N in the soil and retarded its translocation along the soil profile.

## Introduction

Conservation tillage can effectively improve soil quality through the input of fresh organic matter from crop residues [1,2]. This process is recommended worldwide due to increasing concern about the effective utilization of nutrients and the sustainability of agro-ecosystems [3–5]. In these cropping systems, large amounts of crop residue were covered on the soil surface. Considering the high concentration of carbon (C) in crop residue (approximately 40%), the vital role of crop residue in maintaining and improving soil organic C is generally recognized [6], especially in northeastern China [7]. In addition, crop residue alters the turnover of nutrients such as nitrogen (N), phosphorus (P), and potassium (K) in soil-crop systems [8–10]. Maize residue generally contains about 80 kg N ha^-1^ (depending on annual yield and N concentrations), thus acting as an important source contributing to soil N pools [11] and possibly being a potential source of available N for subsequent crop uptake [12,13]. However, the temporal transformation and spatial distribution of crop residue N in soil and the N use efficiency by crops remain unclear. Understanding these processes is important for determining N turnover and crop residue management in agro-ecosystems.

Unlike inorganic N fertilizers, the transformation dynamics and distribution of crop residue N in soil-crop systems are closely linked to crop residue decomposition [14,15]. The rate and process of crop residue decomposition are mainly influenced by the followings: (1) the qualities of the residue, including C/N ratio, lignin, and polyphenol contents [16,17]; (2) the placement of the residue, e.g., incorporation into soil or surface application [18]; and (3) environmental and soil factors such as precipitation, temperature, and biota [13,19,20]. These internal and environmental factors jointly influence the biochemical transformation of crop residue N, and thus alter its temporal and spatial transfer and allocation in soil-crop systems [21,22]. By using an isotope tracing technique (i.e., the crop residue was ^15^N-labeled), the recovery of N derived from incorporated crop residue in soil-crop systems has been studied in both tropical and temperate regions [11,13,20,23]. Unfortunately, the majority of these studies are limited to measuring the recovery of residue N by the first crop following residue application; whereas, the processes and mechanisms of N cycling associated with the release dynamics of crop residue N have not been fully studied [19,22,24], notably in conservation tillage systems.

Because the decomposition of crop residue is time-dependent, clarifying the multi-seasonal recoveries of N derived from the initially applied residue in the soil and crop would lay the foundation for estimating the long-term turnover of residue derived N. Therefore, we conducted a field experiment of two treatments, both of which were applied with ^15^N-labeled maize residue under mulching management in the 1^st^ year. From the 2^nd^ to 4^th^ year, one treatment stopped the application of any maize residue, whereas the soil of the second treatment was re-applied with unlabeled maize residue. Considering the decomposition-controlled N release of the residue, we hypothesized that the initially applied residue N has a relatively long release process, and the sequential application of residue were expected to enhance the immobilization of the initially applied residue N in the soil. This process increases the total recovery of the residue N in soil-crop systems. This experiment would contribute to a better understanding of the dynamic allocation of crop residue N in an agro-ecosystem, and may hold significant implications for developing agronomic management and crop residue application strategies in temperate regions.

## Materials and Methods

### Study site

**Fig 1:**
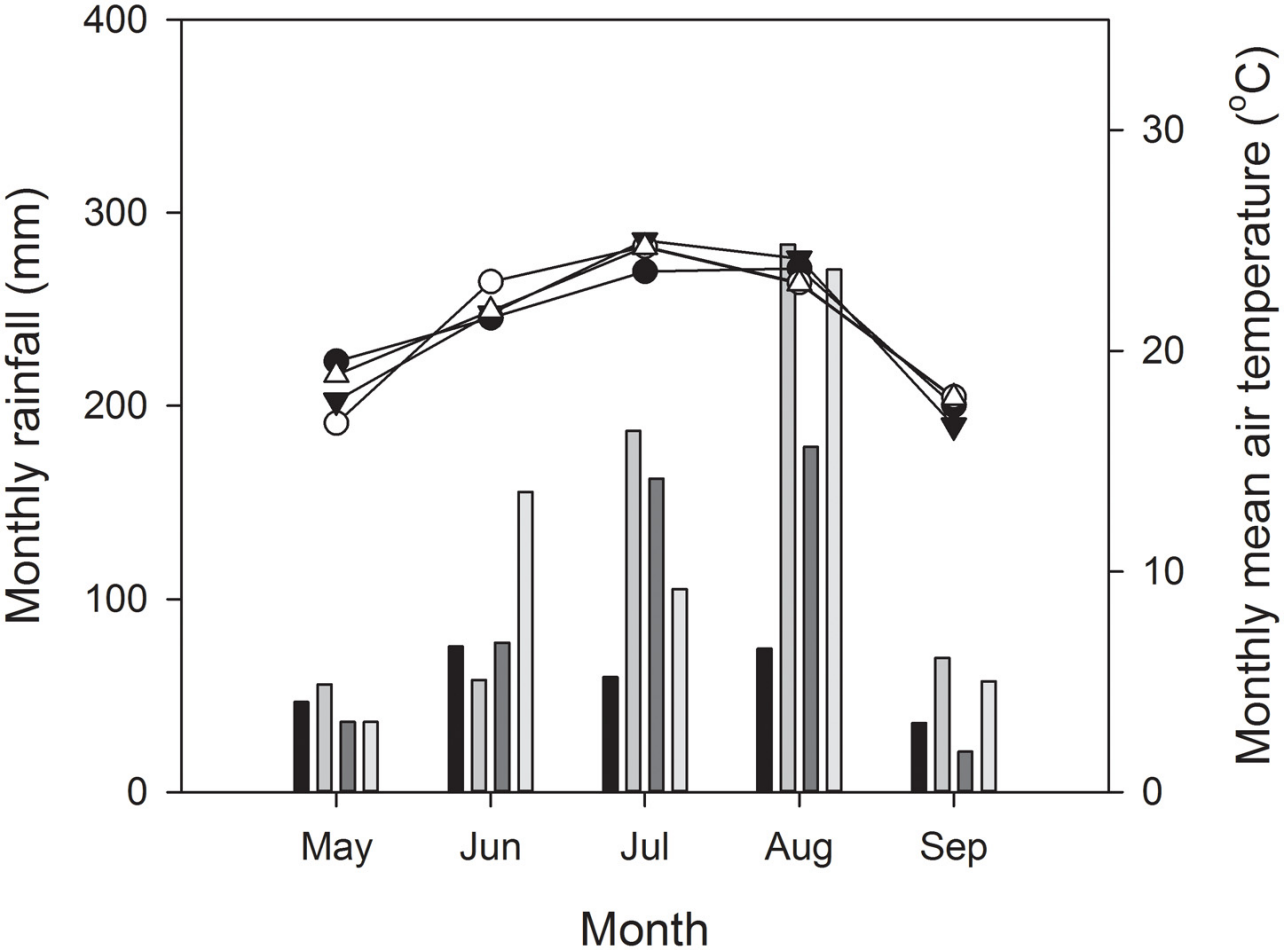
Amount of monthly rainfall and mean air temperature during the 2009 to 2012 growing season.

The field experiment was initiated in the spring of 2009 at the National Field Observation and Research Station of Shenyang Agro-ecosystems (41°32′ N, 122°23′ E) in northeastern China. All necessary permits were obtained from the station for the *in situ* field research. Maize is the staple crop in the typical mono-cropping system, and this crop is sown at the end of April and harvested in late September in this region. The soil type of the experimental field is Alfisol (Typic Hapludoll) [25,26]. The weather at the site is typical of a temperate, humid, continental monsoon climate. During the experimental period (2009–2012), the mean annual precipitation (MAP) was 678 mm (Fig 1). The precipitation during the maize growing season (May-September) accounted for approximately 75% of the MAP. The weather conditions were dry during 2009 growing season (292 mm), but were wet during 2010 and 2012 growing season (654 and 625 mm, respectively). From 2009 to 2012, the temperature during the growing season varied slightly, with an average value of 21.2°C (Fig 1).

### Experiment

The experiment was arranged in a randomized design with three replicates. The plots were randomly arranged in the field with the distance about 2.5 m. The area of each experimental plot was 2.08 m^2^ (1.6 m × 1.3 m) and was surrounded by poly-vinyl-chloride (PVC) boards [25]. The PVC boards were pressed into the soil at a depth of 35 cm and a height of 15 cm above-ground. For all plots, maize was sown at a density of 12 plants per plot. The ^15^N-labeled maize residue was obtained from a ^15^N-labeled fertilization experiment by applying ^15^N-labeled (NH_4_)_2_SO_4_ in 2008. After harvest, the C/N ratio of the maize residue was 51.9 and the weighted ^15^N excess was 31.8%.

Two treatments were included in this study: (1) Treatment 1 (T1), in the 1^st^ year (2009), ^15^N-labeled maize residue was applied on field surfaces after being chopped into 10-cm-long pieces, and from the 2^nd^ to 4^th^ year (2010–2012), no additional maize residue was applied; and (2) Treatment 2 (T2), ^15^N-labeled maize residue was also applied in the 1^st^ year, but from the 2^nd^ to 4^th^ year (2010–2012), unlabeled maize residue was continuously applied as a covering on the soil surface. Both ^15^N-labeleld and unlabeled maize residue was applied at half the yield of maize residue because a portion of the produced residue is generally used for livestock feed or energy use in northeastern China. The application rate was 5.8 Mg ha^-1^ with an annual C input of 2507 kg ha^-1^ and an N input of 48.3 kg ha^-1^.

In this experiment, N fertilizer was applied at a locally moderate rate of 200 kg N ha^-1^ by applying (NH_4_)_2_SO_4_ during the growing season. In addition, P and K fertilizers, as KH_2_PO_4_ and K_2_SO_4_ in the form of pellets, were applied at 30 kg P ha^-1^ and 58 kg K ha^-1^ during the sowing stage for all treatments [25]. Seedbeds for each plot were prepared manually with a reduced disturbance on the soil, and the base fertilizers were incorporated into the topsoil (0–10 cm) prior to maize sowing. After seed sowing, maize residue was applied on the soil surfaces of the field plots. The plots were manually weeded, and the weeds were left on the soil surface of the corresponding plot.

### Soil, plant samplings, and measurements

From 2009 to 2012, soil samples were collected at maize maturity from each plot at depths of 0–10, 10–20, 20–40, and 40–60 cm. Six individual samples at each soil layer were taken with a 3-cm-diameter soil auger and then thoroughly mixed by hand to obtain a composite sample from each plot. The samples were air-dried, and all visible roots and un-decomposed residue was removed and then milled in a rolling drum sieve to < 0.15 mm. As part of the experiment, we also determined the accumulation of KCl-extractable mineral residue N in the deeper layers to estimate the leaching risk of residue N during slower decomposition. Thus we sampled representative fresh soils at depths of 0–20, 20–40, and 40–60 cm for each treatment at maturity in the 3^rd^ (2011) and 4^th^ (2012) year of this experiment. After removing the visible roots and un-decomposed residue, these fresh soils were ground and sieved < 2 mm to prepare to extract mineral N (NH_4_^+^-N and NO_3_^−^-N). The mineral N was measured by the 2 M KCl extraction-MgO-Devarda alloy distillation method [27].

The above-ground maize parts were harvested at maturity manually with a sickle, cutting close to the aerial root. They were separated into stalk, leaf, cob, and grain. Every part was then oven-dried at 65°C to a constant weight to determine the dry-matter content. The representative above-ground samples were milled with a shredder and sieved to < 0.15 mm. The unlabeled maize residue returned to the plots was also prepared to determine the total C and N content.

The biomass of the stubble and root (0–20 cm) of the three plants was selected randomly from each plot at maturity in 2012 for the T1 and T2 treatments. The stubble was severed from the below-ground root with sickles and washed with deionized water. The root, at a depth of 0–20 cm, was removed from the soil within a 30 cm quadrate in which maize was planted in the center and then washed by deionized water on a 1-mm mesh. The stubble and root (0–20 cm) were oven-dried at 65°C to a constant weight. The dry-matter contents of the stubble and root were recorded afterwards. Representative stubble and root samples were ground to pass a 0.15 mm sieve for analyses.

The total N of the soil and plant samples was determined by combustion with an elemental analyzer (Model CN, vario Macro Elemental Analyser System, GmbH, Germany). The ^15^N enrichments of the soil and plant samples and extractable mineral N in the acidified aqueous distillate were measured on an isotope ratio mass spectrometer (Finnigan MAT 251, USA).

### Calculations

For the T1 and T2 treatments, the ^15^N enrichment of each compartment was obtained by subtracting from the corresponding natural ^15^N abundance. The recovery of initially applied residue N was calculated from the amount of residue N recovered in the above-ground crop parts and soils at four depths (0–10, 10–20, 20–40, and 40–60 cm).

#### Weighted ^15^N excess of the crop

Because the N in different maize compartments was not uniformly labeled for each treatment, the weighted ^15^N excess (*WNE*) was used for the maize crop [22]:

$$WNE(\%)=\frac{\sum_{i=1}^{n}atom\%{}^{15}Nexcess_{i}(\%)\times TN_{i}(kgha^{-1})}{\sum_{i=1}^{n}TN_{i}(kgha^{-1})}\times 100$$ (1)

where *i* is a particular plant component, *n* is the total number of plant components including the grain, stalk, leaf, and cob, and *TN (kg ha* ^*-1*^ *)* is the amount of N measured in each crop component, calculated as the product of the concentration of N in the component and its weight. The *WNE* of ^15^N-labeled residue returned in the 1^st^ year (2009) was also calculated using the above Eq (1) for both treatments, but the plant components only included the stalk and leaf.

#### Uptake and recovery of initially applied maize residue N in the crop

The percentage of residue N in the crop total N (*PRNC*) was calculated as follows [28]:

$$PRNC(\%)=\frac{WNE_{crop}(\%)}{WNE_{residueinput}(\%)}\times 100$$ (2)

where *WNE*_*crop*_ *(%)* is the ^15^N enrichment of the crop above-ground components (including the grain, stalk, leaf, and cob) calculated by Eq (1) and *WNE*_*residue input*_ *(%)* is the ^15^N enrichment of the returned residue.

The amount of residue N taken up by the crop (*ARNC*) was calculated as follows [15]:

$$ARNC(kg\;ha^{-1})\;=\;PRNC(\%)\;\times \;CTN(kg\;ha^{-1})$$ (3)

where *CTN (kg ha* ^*-1*^ *)* is the total amount of N measured in the crop above-ground biomass.

The annual recovery of residue N in the crop (*RRNC*) was calculated as the following:

$$RRNC(\%)=\frac{ARNC(kgha^{-1})}{TN_{input}(kgha^{-1})}\times 100$$ (4)

where *TN*_*input*_ *(kg ha* ^*-1*^ *)* is the total amount of residue N applied in the 1^st^ year.

#### Content and recovery of initially applied maize residue N in the soil

The percentage of residue N in the soil total N (*PRNS*) was calculated as the following:

$$PRNS(\%)=\frac{atom\%{}^{15}Nexcess_{soil}(\%)}{WNE_{residueinput}(\%)}\times 100$$ (5)

where *atom%* ^*15*^ *N excess*_*soil*_ *(%)* is the soil ^15^N enrichment in each layer (0–10, 10–20, 20–40, and 40–60 cm).

The content of residue N in the soil (*CRNS*) was calculated as the following [15]:

$$CRNS(kg\;ha^{-1})\;=\;PRNS(\%)\;\times \;STN(kg\;ha^{-1})$$ (6)

where *STN (kg ha* ^*-1*^ *)* is the total N content in each soil layer (0–10, 10–20, 20–40, and 40–60 cm), calculated as the product of the concentration of N in the compartment and its weight. The weight of each soil layer calculated through multiplying the volume by the corresponding bulk density. The soil bulk density is 1.17, 1.44, 1.45 and 1.38 g cm^-3^ at depths of 0–10, 10–20, 20–40, and 40–60 cm, respectively.

The recovery of residue N in the soil (*RRNS*) in the sampling year was calculated as the following:

$$RRNS(\%)=\frac{CRNS(kgha^{-1})}{TN_{input}(kgha^{-1})}\times 100$$ (7)

where *TN*_*input*_ *(kg ha* ^*-1*^ *)* is the total amount of residue N applied in the 1^st^ year.

#### Total recovery of initially applied maize residue N in the soil-crop

The cumulative recovery of residue N in the soil-crop (*CRRNSC*) was calculated as the following:

$$CRRNSC_{j}(\%)\;=\;\sum_{i=1}^{j}RRNC_{j}(\%)\;+\;\sum_{k=1}^{4}RRNS_{kj}(\%)$$ (8)

where $\sum_{i=1}^{j}RRNC_{j}$ is the cumulative recovery of residue N in the crop from the *i* ^th^ to *j* ^th^ year, $\sum_{k=1}^{4}RRNS_{kj}$ is the total recovery of residue N in the soil in the *j* ^th^ year, *k* is a particular soil layer, *4* is the total number of soil layers including 0–10, 10–20, 20–40, and 40–60 cm.

### Statistical analysis

A statistical analysis was performed using the software package SPSS 13.0 for Windows (SPSS Inc., Chicago, IL, USA). Repeated-measure analysis of variance (ANOVA) was used to examine the differences in crop yield, total and residue N uptake, soil total N, residue N contents, and residue N recovery in the soil-crop systems with time between treatments. Two-way ANOVA was conducted to compare the combined effects of the soil layer and residue treatment on the total and residue-derived extractable mineral N. The data were then analyzed separately by residue treatment, sampling year and soil layer using a series of independent-sample t-tests or one-way ANOVAs. Multiple comparisons were performed based on the least significance difference (LSD) test at a 95% confidence level.

## Results

### Multi-seasonal uptakes of total N and initially applied maize residue N in the crop

#### Above-ground biomass and uptakes of total N and residue N in the crop

The above-ground crop yields exhibited no significant difference between the T1 and T2 treatments in the identical year, although the yields varied significantly throughout the experiment years (Table 1). The highest crop yield occurred in the 3^rd^ year for both treatments because of favorable precipitation. However, in the 1^st^ and 2^nd^ year, the crop yields were much lower as a result of extremely dry and wet conditions, respectively, during the growing season (Table 1 and Fig 1). For the T1 and T2 treatments, the total N uptake in the above-ground crop parts, ranging from 208 to 327 kg ha^-1^ and from 209 to 316 kg ha^-1^, respectively, was also significantly affected by precipitation. The total N uptake by the crop was at a lower level in the 1^st^ and 2^nd^ year, and the biggest uptake occurred in the 3^rd^ year (Table 1). For both treatments, the total N uptake exhibited no significant difference throughout the experiment (*p* = 0.204).

**Table 1:** Above-ground biomass and N uptake in the crop under different treatments from 2009 to 2012.

Treatment	Year	Biomass (Mg ha^-1^)	Total N uptake (kg ha^-1^)	Residue N uptake (kg ha^-1^)	Percentage of residue N in total N uptake (%)	Recovery of residue N (%)
**T1**	**2009**	**20.4±1.10**	**215.5±14.23**	**1.38±0.11**	**0.6±0.1**	**3.2±0.2**
	**2010**	**21.5±0.97**	**207.9±11.22**	**2.83±0.29**	**1.4±0.2**	**6.5±0.2**
	**2011**	**28.8±2.08**	**326.5±33.19**	**3.10±0.23**	**1.0±0.1**	**7.1±0.1**
	**2012**	**25.8±0.95**	**238.8±8.96**	**1.58±0.15**	**0.7±0.2**	**4.1±0.3**
**T2**	**2009**	**19.3±1.05**	**209.0±12.97**	**1.45±0.11**	**0.7±0.1**	**3.0±0.2**
	**2010**	**21.3±1.91**	**210.4±15.53**	**3.16±0.32**	**1.5±0.2**	**6.5±0.3**
	**2011**	**27.9±2.02**	**316.3±23.96**	**2.03±0.21**	**0.6±0.1**	**4.2±0.2**
	**2012**	**27.6±1.17**	**253.1±18.71**	**2.13±0.13**	**0.8±0.1**	**4.4±0.3**

T1: single residue application; T2: annual residue application (the same below). Values reported as mean ± standard deviations, n = 3.

After the 1^st^ growing season, the uptake of initially applied residue N by the above-ground crop parts was 1.42 kg ha^-1^ and only accounted for less than 0.7% of the corresponding total N uptake. During the subsequent seasons, the residue N uptake by crops in the T1 treatment increased to the largest value (3.10 kg ha^-1^) in the 3^rd^ year, whereas decreasing significantly afterward (Table 1). The largest uptake of initially applied residue N in the T2 treatment (3.16 kg ha^-1^) occurred in the 2^nd^ year; the amount of residue N in the crop then displayed a decrease but remained unchanged during the last two years. For the T1 and T2 treatments, the largest contribution of residue N to total N uptake was found in the 2^nd^ year and reached 1.4% and 1.5% of the corresponding total N uptake (Table 1), respectively.

#### Recovery of residue N in the crop

In the 1^st^ year, the crop above-ground parts recovered, on average, 3.1% of the initially applied residue N (Table 1). In the T1 treatment, the recovery increased gradually to the largest value (7.2%) in the 3^rd^ year, but decreased significantly to 4.1% in the 4^th^ year. For the T2 treatment, the recovery increased to the greatest value (6.5%) in the 2^nd^ year, and then remained unchanged at an average level of 4.3% (Table 1). Over the four years, the cumulative recovery in the crop for the T1 treatment (20.9%) was higher than that of the T2 treatment (18.2%).

#### Biomass, N uptake, and recovery of residue N of the root-stubble

In the T1 and T2 treatments, the biomass and N uptake of the root-stubble were much lower than that in the above-ground parts in the 4^th^ year (Tables 1 and 2), showing no significant treatment effect (*P* = 0.695 and 0.631, respectively). The root-stubble recovered, on average, 0.4% of the initially applied residue N with no significant difference between the treatments (*P* = 0.857). In addition, the recovery of the residue N in root-stubble accounted for only approximately 10% of that recovered in the above-ground parts in the 4^th^ year (Tables 1 and 2).

**Table 2:** Biomass, N uptake, and residue N recovery of root-stubble under different treatments at maturity in the 4^th^ year (2012).

Treatment	Biomass (Mg ha^-1^)	Total N uptake (kg ha^-1^)	Residue N recovery (%)
**T1**	**2.27±0.13**	**13.28±1.11**	**0.4±0.1**
**T2**	**2.47±0.15**	**14.35±1.33**	**0.4±0**

Values reported as mean ± standard deviations, n = 3.

### Total N and initially applied maize residue N in the soil

#### Changes in total N in the soil

**Fig 2:**
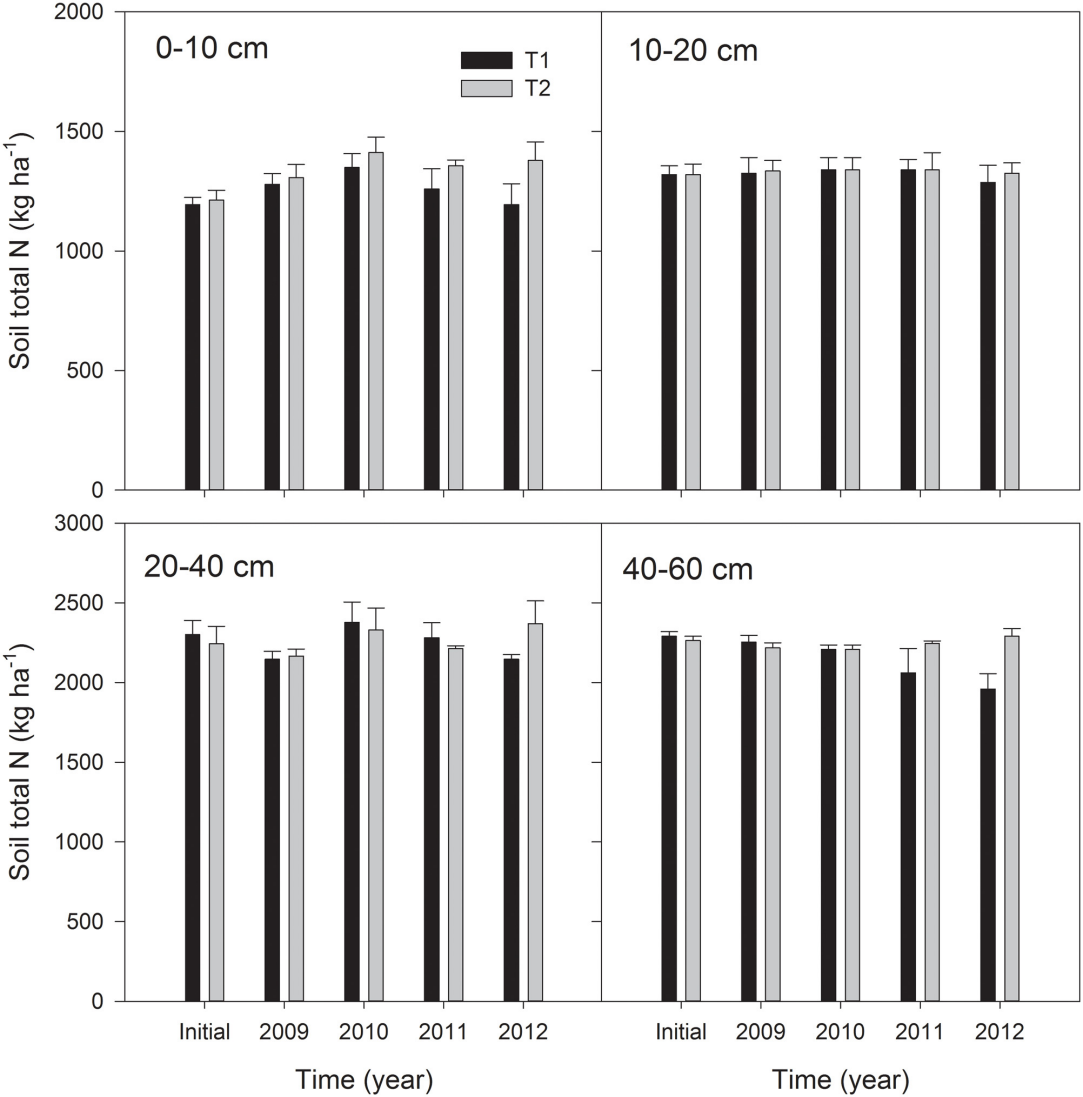
Changes in soil total N content for four soil layers (0–10, 10–20, 20–40, and 40–60 cm) under different treatments during 2009 to 2012. Error bars mean standard deviations, n = 3.

For the T1 treatment, the total N in the topsoil increased gradually to the largest value (1349 kg ha^-1^) in the 2^nd^ year (2010), then decreased gradually to the initial level (1194 kg ha^-1^) in the 4^th^ year (Fig 2). Continuous application of residue (T2 treatment) led to a significant accumulation of total N in the topsoil (0–10 cm) in the first two years and the total N content remained relatively stable thereafter (on average, an increase of 14.0% compared with the initial value). The total N content in the deeper layers (10–60 cm) for the T1 treatment exhibited an obvious decline in the 4^th^ year, whereas remaining unchanged for the T2 treatment throughout the experiment (Fig 2).

#### Changes in residue N in the soil

**Fig 3:**
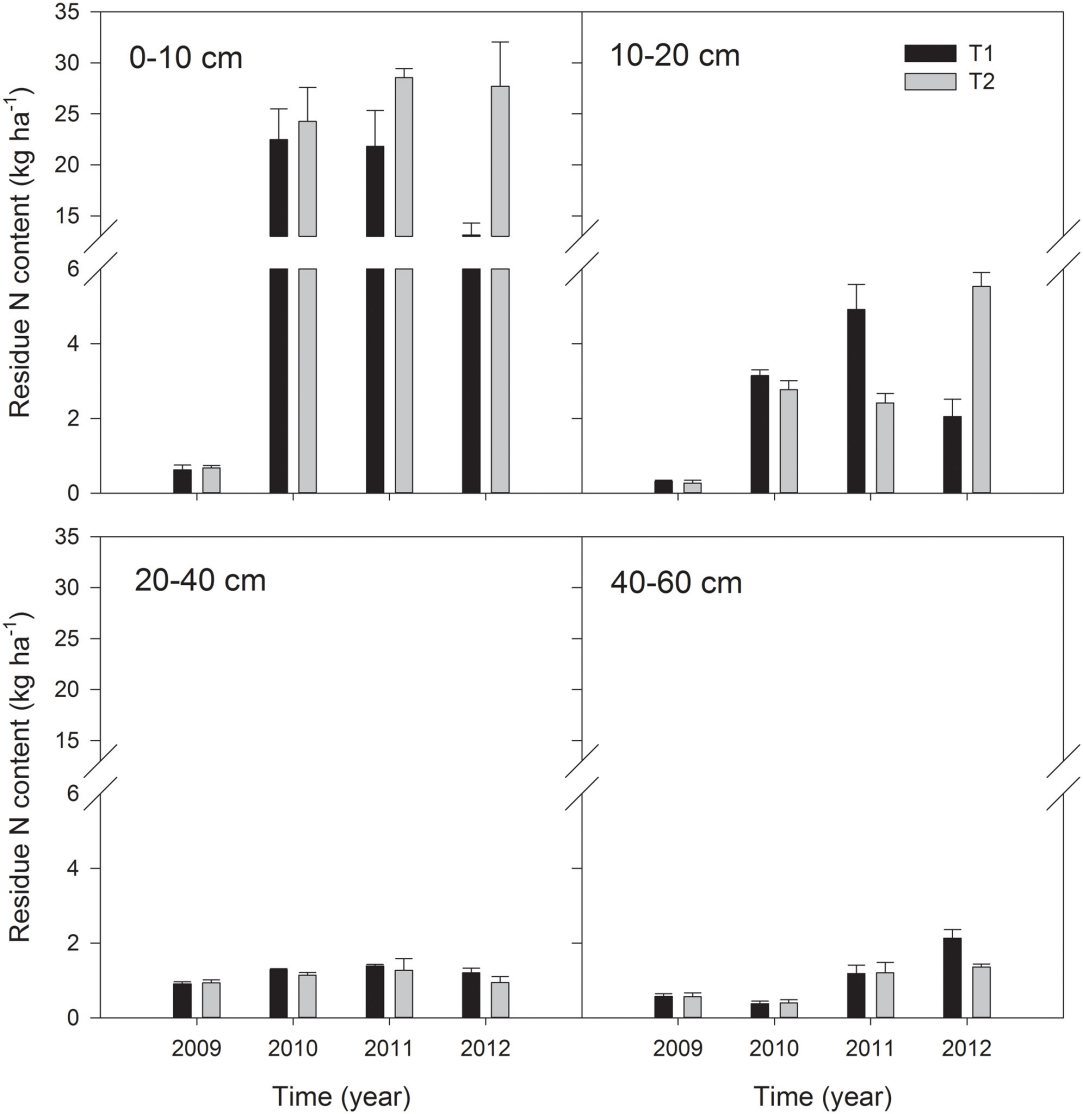
Changes in the residue N content for the four soil layers (0–10, 10–20, 20–40, and 40–60 cm) under different treatments during 2009 to 2012. Error bars indicate standard deviations, n = 3.

In the 1^st^ year (2009), only a small amount of initially applied residue N was incorporated into the soil total N, which accounted for no more than 1% of the soil total N increment (Fig 3). In the T1 treatment, the residue N content in the topsoil increased significantly to the highest level during the 3^rd^ to 4^th^ year, and the contribution of the residue N to soil total N displayed its highest value of 1.7% in the 4^th^ year, and then decreased substantially afterwards (Fig 3, *p* = 0). In the subsoil (10–20 cm), the residue N content was relatively lower than in the topsoil, whereas exhibiting a similar variation trend as in the topsoil. In the deeper layers (20–60 cm), the residue N content was much lower compared to the topsoil, but exhibited an obvious increase over time in the 40–60 cm layer. For the T2 treatment, the residue N content in the topsoil increased gradually to the highest level in the 3^rd^ to 4^th^ year (Fig 3), and, on average, contributed 2.1% of the soil total N. In the subsoil, the residue N content increased gradually throughout the experiment. However, the deeper layers (20–60 cm) generally contained lower residue N and remained relatively stable (Fig 3).

#### Recovery of residue N in the soil

**Fig 4:**
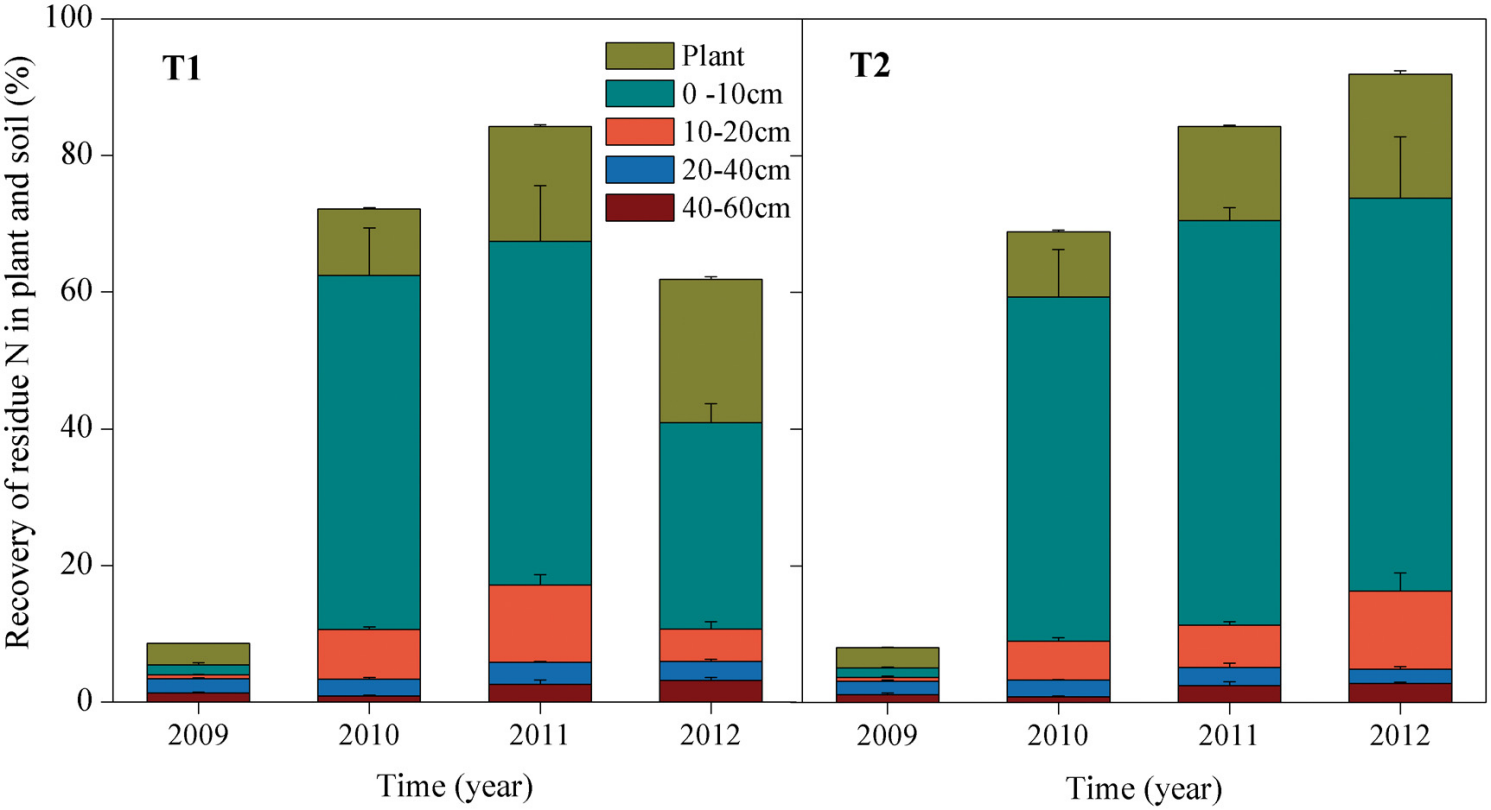
Cumulative recovery of residue N in soil and crop under different treatments during 2009 to 2012. Error bars indicate standard deviations, n = 3.

In the entire soil depth (0–60 cm), the recovery of initially applied residue N was approximately 5% in the 1^st^ year, whereas it increased significantly in the 2^nd^ year in both treatments (Fig 4). For the T1 treatment, the recovery increased to its largest value (67.4%) in the 3^rd^ year but then displayed a significant decrease to 40.9% at the last sampling (2012). However, for the T2 treatment, the total recovery increased gradually to its largest value of 73.8% at the last sampling (Fig 4). The recovery of the residue N in each soil layer was significantly different throughout the experiment (*P* = 0). The topsoil recovered the vast majority of the residue N from the 2^nd^ to 4^th^ year. The deeper layers (10–60 cm) recovered relatively smaller amounts of the residue N, but total recovery of the residue N in the deeper layers increased gradually with time for both treatments. At the last sampling, the residue N recovery in the T1 treatment decreased sharply in the topsoil and subsoil but displayed a significant increase in the 40–60 cm layer (Fig 4). For the T2 treatment, the recovery remained unchanged in the topsoil when compared to that in the T1 treatment, whereas increasing significantly in the subsoil in the 4^th^ year.

### Total and initially applied maize residue derived extractable mineral N in the soil

In 2011 and 2012, the contents of total extractable mineral N in the T1 treatment were generally higher than that in the T2 treatment (Table 3), thus the percentage of extractable mineral N in the soil total N in the T1 treatment was much higher than that of the T2 treatment along the soil profile. In both sampling years, the T1 treatment resulted in higher contents of initially applied residue derived mineral N than the T2 treatment along the soil profile (Table 3), notably of NO_3_^−^ (13.5–71.3 μg kg^-1^ vs. 4.9–38.3 μg kg^-1^). For both treatments, the proportion of the mineral residue N in the soil total residue N in the 0–20 cm layer was significantly lower than that in the 20–40 cm and 40–60 cm layers (Table 3). In terms of treatment effects, the T1 treatment generally led to much higher proportions of mineral residue N in the soil total residue N than the T2 treatment.

**Table 3:** Contents of soil total mineral N (TMN) and residue mineral N (RMN), proportions of the total mineral N in soil total N (TMN/TN), and the mineral residue N in soil total residue N (RMN/RN) in the soil profile under different treatments in 2011 and 2012.

Treatment	Depth (cm)	TMN (mg kg^-1^)		RMN (μg kg^-1^)		TMN/TN (%)		RMN/RN (%)	
		2011	2012	2011	2012	2011	2012	2011	2012
**T1**	**0–20**	**21.45±0.90**	**16.76±1.02**	**70.4±8.82**	**80.8±8.92**	**2.1±0.2**	**1.9±0.1**	**0.7±0.1**	**1.5±0.2**
	**20–40**	**14.18±1.12**	**11.79±1.11**	**52.0±0.51**	**32.3±4.09**	**1.8±0.2**	**1.6±0.1**	**29.1±3.3**	**38.5±5.3**
	**40–60**	**14.86±0.89**	**11.99±0.85**	**36.2±1.11**	**37.7±0.86**	**2.0±0.2**	**1.7±0.2**	**57.2±4.5**	**4.9±0.6**
**T2**	**0–20**	**19.27±1.76**	**15.20±0.73**	**52.5±9.09**	**18.1±1.75**	**1.9±0.1**	**1.5±0.1**	**0.4±0.1**	**0.1±0**
	**20–40**	**9.84±0.94**	**10.59±0.44**	**24.7±3.62**	**9.6±0.77**	**1.3±0.1**	**1.3±0.1**	**5.8±0.9**	**2.8±0.6**
	**40–60**	**11.46±0.46**	**11.62±0.32**	**27.2±1.87**	**12.8±0.90**	**1.4±0.1**	**1.4±0.1**	**4.6±0.8**	**2.6±0.1**

Values reported as mean ± standard deviations, n = 3.

### Cumulative recovery of initially applied maize residue N in the soil-crop systems

In the 1^st^ year (2009), only a small portion (on average, 8.4%) of initially applied residue N was recovered in the soil-crop systems (Fig 4). However, since the 2^nd^ year, the cumulative recovery displayed a significant increase, which was mostly attributed to the residual N recovery in the soil (Fig 4). No significant treatment effect of the cumulative recovery of the residue N in the soil-crop systems was noted until the 4^th^ year. In the 4^th^ year, the cumulative recovery decreased greatly (to 61.9%) in the T1 treatment, whereas the recovery increased gradually to its largest value of 91.9% in the T2 treatment (Fig 4). These differences were mainly ascribed to the changes of the residue N in the soil.

## Discussion

### Uptake of initially applied maize residue N in the crop

In conservation tillage systems, recycled crop residue serves as an important source of organic N [22,29]. Different to the high availability of fast-acting fertilizers such as chemicals and manure N for crop utilization, most residue N is in polymeric forms such as proteins or peptides [30]. The residue N can be released gradually during decomposition and mineralization and is ultimately involved in N cycling [22,31]. However, the decomposition of residue and the crop uptake of the released N varied greatly because of the different quality and quantity of the residue, climate conditions (temperature and precipitation), and debris treatment in the field [15,32].

When crop residues with relatively high C/N ratios (> 30) were incorporated into soil, between 4% and 13% of the applied residue N were reported to be recovered by crops in the 1^st^ growing season regardless of regions [22,24,32,33]. Whereas, the crop residue applied on the soil surfaces generally undergoes slower decomposition than those incorporated into the tillage layer because of limited microbial accessibility, notably under lower temperature and precipitation conditions [14,18,29], thus resulted in a lower crop uptake of residue N in the 1^st^ year in our experiment (Table 1). However, the remarkably improved cumulative recoveries of the residue N in the crop during the 2^nd^ to 4^th^ year were significantly larger than that obtained in the tropics (15% vs. 6–7%), suggesting that the prolonged nutrient release enhanced the sustainable residue N uptake by the sequential plant, eventually favoring cumulative crop uptake of the residue N (18–21%) than those obtained in other comparable multi-seasonal studies (12–16%) [11,14,29,34].

When there was no subsequent residue application from the 2^nd^ year, negligible amounts of visible residue remained on the field surface after three years, implying significantly less N released from the initially applied residue afterwards. In addition, the accumulation of the residue N in subsequently growing root-stubbles was limited for N supply. Thus, the recovery of the residue N in the crop peaked in the 3^rd^ year whereas declined significantly in the 4^th^ year. Comparatively, sequential application of crop residue significantly decreased the crop utilization of the initially applied residue N in the 3^rd^ year in spite of the unchanged N uptake, suggesting the altered N sources for crop uptake (e.g., the subsequently applied maize residue and native available N in the soil). However, the enhanced retention of the residue N in soil during the experimental periods indicated that the decomposition of the initially applied maize residue was not retarded. Instead, enhanced microbial immobilization of the residue N by the stimulated microbial growth led to the decreased crop uptake of the residue N in the T2 treatment [14,16].

### Dynamics of initially applied maize residue N in the soil

During the entire sampling interval, the accumulated amount of the residue N in the soil remained significantly larger than in the crop for both treatments (Figs 3 and 4), indicating a dominant retention of decomposed residue N in the soil, possibly as potential N sources for sustainable soil N maintenance and crop utilization [15]. In previous studies with the residue incorporated into the soil, the maximum accumulation of the residue N in the soil occurred more rapidly than that applied on the soil surface due to faster decomposition [11,14,15,18,22,34]. This accumulation decreased sharply afterwards because of the significant loss either via leaching or denitrification induced by inorganic N accumulation [10,12,22,32,33]. Comparatively, in our treatments, the accumulated amount of initially applied residue N in the soil was significantly greater over several seasons, indicating that the surface application of residue with slower decomposition was favorable for the stabilization and accumulation of the residue N in soil.

Because the maize residue was applied on the soil surfaces in this experiment, the topsoil (0–10 cm) was likely to contain more products of decomposed residue than the deeper layers. However, the increased retention of the residue N in the deeper soils with time indicated the significantly vertical translocation of decomposed residue N along the soil profile [21]. For the single application of the residue, the unchanged residue N content in 0–10 cm depth during the 2^nd^ to 3^rd^ year was likely attributed to the gradual N release during the residue decomposition, potentially offsetting the vertical translocation and possible plant uptake of residue derived N in the topsoil. After three years, however, the lack of supplemental residue N resulted in a significant decrease of the residue N in the topsoil [14,35]. Comparatively, the sequential application of residue substantially increased the retention of initially applied residue N in 0–60 cm soil profile throughout the four years, especially favored the accumulation of the residue N on the topsoil because of hotspot for microbial immobilization of the residue N [14,16].

The vertical translocation dynamics of the residue N is not simply dependent on its concentration in soil, but more importantly, the translocation extent was essentially associated with the accumulation of extractable mineral residue N, notably NO_3_^−^-N [12,22,32]. In the T1 treatment, the higher contents of mineral residue N and proportions of the mineral residue N in soil total residue N along the soil profile might, to an extent, result in a higher loss risk of the residue N and lead to a lower recovery of the residue N in the soil in the 4^th^ growing season when experiencing a higher rainfall (Fig 1). The sequential application of residue significantly decreased both extractable mineral residue N and its proportion in the accumulated soil total residue N (Table 3). Thus, the mineralization of the initially applied residue N was restrained, i.e., significant amounts of residue N was likely preserved in more stable forms, notably in the topsoil [14].

### Mechanisms of residue and total N retention in the soil-crop systems

In agro-ecosystems, residue N can be taken up by crops, retained in soil, and/or lost from the soil-crop systems by leaching and gas emission [14]. Our experiment with residue applied on soil surface presented different annual dynamics and cumulative recoveries for residue N in the soil-crop systems (Fig 4), in comparison to previous studies with residue incorporated into the soil. The significantly larger cumulative recovery of residue N in the soil and above-ground plant components (85%-92% vs. 58%-75%) indicated that a slower decomposition of surface-leaving crop residue can significantly improve the total recovery of the residue N, notably via gradual accumulation in the soil [34]. The sequential application of residue, concomitantly with inorganic N application, was favorable for multi-seasonal recovery of the initially applied residue N (92% vs. 62% during four years) in soil-crop systems, mainly because of the enhanced immobilization and re-utilization of the residue N by activated soil microorganisms [10,33].

Conservation tillage with crop residue return is widely accepted to be able to increase the topsoil total N content. In our experiment, simultaneously with the augmentation of the residue N in the topsoil, the unlabeled soil N increased significantly, indicating that the accumulation of soil total N along with the residue decomposition was attributed to both the immobilization of fertilizer N and the transformation of the residue N [9,14,22]. However, the lacking of subsequent application of fresh residue decreased the total N in topsoil at the last sampling, mainly because of the less microbial N immobilization and the mineralization of previously accumulated soil organic N [10,33]. Comparatively, the re-applied crop residue favored the maintenance of the total N in the topsoil throughout the experiment period and decreased both residue derived and total soil mineral N (Table 3), clearly indicating the stabilization of soil N (including residue and fertilizer derived N and the native portion in soil) manipulated by available carbon derived from continuous maize residue input [10,35]. Therefore, the sequential application of residue was an essential practice to reduce the leaching risk for both initially applied residue derived and soil total N [36,37].

## Conclusions

Based on a four-year micro-plot ^15^N-tracing experiment, this study explored the dynamic allocation of maize residue N in a soil-crop system which was critically controlled by decomposition. Maize residue applied on soil surfaces released small amounts of N in the 1^st^ year, and the majority of the residue N tended to be released during subsequent years. With residue decomposition, significantly higher amounts of the residue N were retained in the soil than was taken up by the crop. Compared to a single residue input, the sequential application of residue could enhance the retention of the initially applied residue N in the soil profile, thus significantly improving the cumulative recovery in the soil-crop systems. More importantly, the sequential application of maize residue improved the stability of the residue N and total N, which was favorable to reduce the leaching loss risk of the soil N. Therefore, the residue under conservation tillage practices is of great significance to improving the quantity and quality of soil N in a temperate agro-ecosystem.
